# Numerical Simulation of Reversed Austenite Evolution during Intercritical Tempering of Low-Carbon Martensitic Stainless Steel

**DOI:** 10.3390/ma17071476

**Published:** 2024-03-24

**Authors:** Dingpeng Huo, Jielong Peng, Xiangjun Chen, Shenghua Zhang

**Affiliations:** 1 State Key Laboratory of Featured Metal Materials and Life Cycle Safety for Composite Structures, MOE Key Laboratory of New Processing Technology for Nonferrous Metals and Materials, School of Resources, Environment and Materials, Guangxi University, Nanning 530004, China; 18271934318@163.com; 2 Zhuhai Lingda Compressor Co., Ltd., Zhuhai 519100, China; 18852867172@163.com; 3 Shenyang National Laboratory for Materials Science, Institute of Metal Research, Chinese Academy of Sciences, Shenyang 110016, China; xjchen@imr.ac.cn; 4 Key Laboratory of High Performance Structural Materials and Thermo-Surface Processing, Education Department of Guangxi Zhuang Autonomous Region, Guangxi University, Nanning 530004, China

**Keywords:** 00Cr13Ni4Mo steel, reversed austenite, JMAK equation, thermal stability

## Abstract

Since the formation of reversed austenite during critical tempering treatment is an important factor affecting the mechanical properties of 13Cr4Ni martensitic stainless steel, a detailed study of the content and morphology of reversed austenite in heat treatment is needed. In this study, the variation curves of a reversed austenite volume fraction with holding time at different tempering temperatures were measured by in situ X-ray diffraction (XRD), and the reversed austenite and carbides of each process were evaluated by transmission electron microscopy (TEM). The austenite content shows a parabolic change with the increase in the tempering temperature; the maximum can reach a peak of about 6.8% at 610 °C, and drops to 0% at 660 °C. It also shows a parabolic change with the extension of the holding time, reaching a maximum of about 9.2% at 5 h of holding time, and a decreasing trend at 10 h of holding time, about 6.8%. The results show that the precipitation of carbides in the microstructure causes elemental segregation at grain boundaries and inside, which is one of the main factors affecting the thermal stability of reversed austenite formation. The kinetic process of reversed austenite during the tempering process was simulated using the JMAK model and the KM model, which can describe the trend of reversed austenite content during the tempering process. Combining the two models, a mathematical model for the room-temperature reversed austenite content under different processes was obtained, and this can predict the room-temperature austenite content.

## 1. Introduction

Low-carbon martensitic stainless steels with excellent comprehensive mechanical properties and an adjustable structure are widely used in many fields such as petroleum pipelines and hydraulic turbines [1,2,3,4,5]. With the further development of energy resources in extreme environments such as the seabed and the Arctic, higher requirements are put forward for the performance of low-carbon martensitic stainless steels [6,7]. Low-carbon martensitic stainless steels with a microstructure consisting of hard martensite lath embedded in soft and ductile reversed austenite possess an outstanding combination of strength and formability. The mechanical properties of these steels are highly dependent on their microstructural characteristics, such as volume fraction, morphology, and distribution of constituents, which, in turn, are determined by the reversed austenite condition at the intercritical temperature. Because of the significant influence on the final microstructure and mechanical properties of low-carbon martensitic stainless steels, the formation of reversed austenite in the intercritical temperature range has been widely studied in recent decades [8,9,10].

Much effort has been invested in introducing some retained austenite into bainite or martensite steels via well-designed heat treatments, such as quenching–partitioning [11], quenching and tempering [12], quenching and modest tempering [13], and quenching and intercritical tempering [14]. During the intercritical tempering, austenite dispersed at the original austenite grain boundary and between the martensitic laths has enriched a large number of stability elements, so that it can remain stable during cooling and be retained at room temperature [15]. Studies have indicated that the amount of reversed austenite increases with an increase in the holding time initially and then becomes constant [14]. This phenomenon seemed to have occurred because the amount and chemical compositions of reversed austenite are not expected to change during the isothermal holding step of intercritical tempering when martensite and austenite are in thermodynamic equilibrium at a given tempering temperature. Therefore, it is generally agreed that the thermal stability of reversed austenite, which is determined by the chemical composition, can be retained. Consequently, the amount of retained austenite at room temperature was maintained [14]. This viewpoint has been accepted widely and some heat treatments have been designed accordingly. However, some large Fe-13%Cr-4%Ni steel castings have always showed poor ductility and toughness after intercritical tempering for a long duration. Microstructural analyses [16] have revealed that the amount of reversed austenite in these castings is much lower than expected, implying that the amount of reversed austenite decreases after extended isothermal holding during intercritical tempering. Extended isothermal holding leads to a decrease in the thermal stability of some austenite, resulting in its transformation into martensite during the cooling process, which is easily overlooked.

Since several processing parameters, including the heating rate, intercritical temperature, holding time, and cooling rate, are usually variable in an industrial production line, many research works have been conducted to evaluate the effects of these parameters on austenite formation during different stages of processing [16,17,18]. Han et al. [17] found that the heating rate during tempering has an impact on the morphology of cold rolled reversed austenite in Fe-(5-9) Mn-0.05C steel. Song et al. [18] pointed out that the austenite content first increases and then decreases to a stable state along with the increase in the intercritical tempering temperature. Zhang et al. [16] found that the austenite content exhibits a parabolic characteristic of first increasing and then decreasing with the extension of the holding time at a constant tempering temperature. Although many investigations have been performed so far with clearly presented experimental and modeling results for the formation of reversed austenite, it seems that more studies need to be conducted with regard to the influence of tempering parameters on the formation kinetics of reversed austenite in these steels, especially for tempering cooling, as these parameters control the steel microstructure during the intercritical tempering stage. 

In the present research work, a detailed study is conducted to evaluate the effect of intercritical tempering process parameters, i.e., the tempering temperature, tempering time and cooling rate, on the formation kinetics of reversed austenite in 00Cr13Ni4Mo steel. Moreover, the Johnson–Mehl–Avrami–Kolmogorov (JMAK) model [19,20] and modified H-M model [21] have been used for modeling the kinetics of austenite formation for intercritical tempering process parameters in both the intercritical tempering isothermal stage and cooling stage. The evolution of reversed austenite in the steel microstructure with carbides under different tempering processes was also evaluated.

## 2. Experimental Materials and Methods

The 00Cr13Ni4Mo steel (Shenyang Institute of Metals, Shenyang, China) used in this paper was melted in a vacuum high-frequency induction melting furnace, and then forged into a square bar with a size of 300 mm × 150 mm × 150 mm in the temperature range of 1050~1150 °C, and its chemical composition was tested, as shown in Table 1.

**Figure 1:**
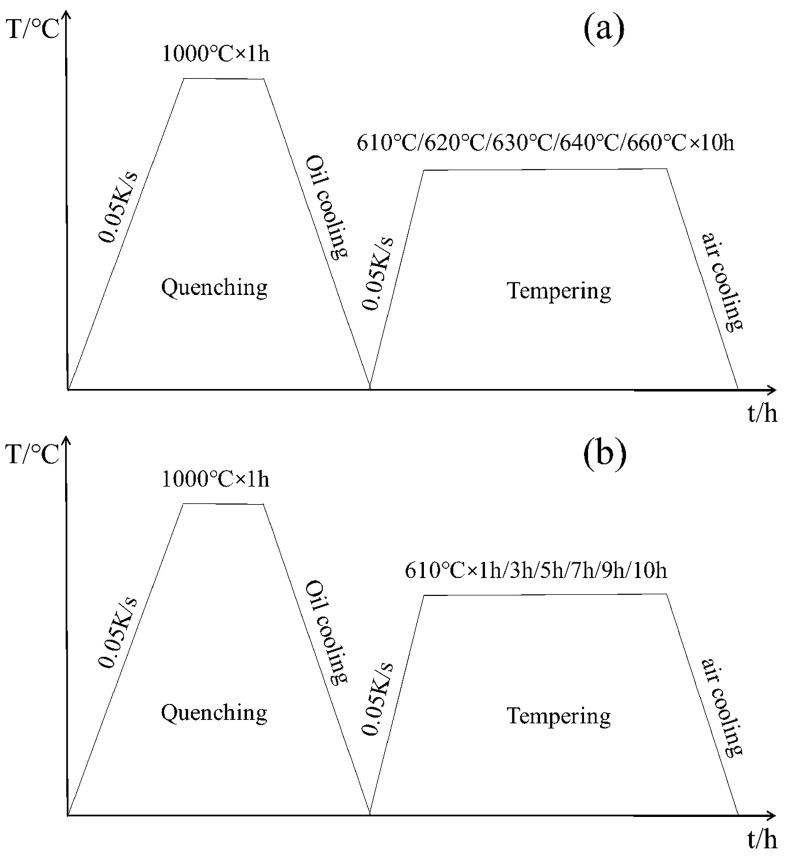
The heat treatment flow chart under different tempering temperatures and holding times. (**a**) Holding at different tempering temperatures for 3 h; (**b**) Holding at 610 °C for different times.

**Figure 2:**
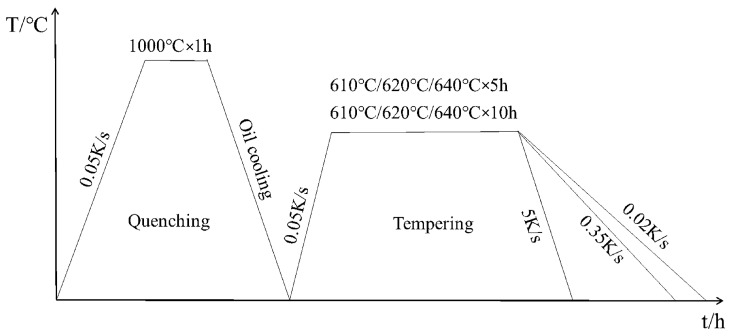
Flow chart of heat treatments at different cooling rates.

The square rod with a size of 60 mm × 10 mm × 10 mm was normalized in a box-type resistance furnace at 1000 °C for 1 h, and then oil cooled to obtain single-phase martensite lath [22]. Then, the sample was divided into three groups. The first group was processed into 10 mm × 10 mm × 0.5 mm thin slices by wire cutting and tempered at 610 °C, 620 °C, 630 °C, 640 °C and 660 °C for 10 h, respectively, as shown in Figure 1a. Finally, the sample was cooled in air to room temperature. The second group was processed into 10 mm × 10 mm × 5 mm bulk samples by wire cutting, tempered and held for 1 h, 3 h, 5 h, 7 h, 9 h and 10 h, and then air cooled to room temperature, as shown in Figure 1b. The third group of samples were made into 10 mm × 10 mm × 5 mm block samples and Φ3 mm × 10 mm rod samples by wire cutting. The block samples were held at 610 °C, 620 °C and 640 °C for 5 h and 10 h, respectively, and cooled to room temperature at 0.02 K/s, 0.35 K/s and 5 K/s, as shown in Figure 2. To measure the initial (*A*_S_) and end (*A*_f_) temperatures of austenite transformation, as well as the initial (*M*_S_) and end (*M*_f_) temperatures of martensitic transformation, the rod-like sample was subjected to thermal expansion experiments (L78 RITA) under the same processes in Figure 2.

To calculate the content of reversed austenite in the process of tempering [23], in situ X-ray diffraction patterns (XRD) of the samples during tempering were measured by a Bruker D8 advance diffractometer. Furthermore, the X-ray diffraction patterns of all the samples after tempering at room temperature were also measured. To further explore the factors affecting the thermal stability of reversed austenite, transmission electron microscopy (TEM, FEI TECNAI G2 F30, FEI USA, Inc.) was used to characterize the microstructure under different heat treatment processes. 

## 3. Results and Discussion

### 3.1. The Kinetic Curves of Reversed Austenite for Different Tempering Processes

**Figure 3:**
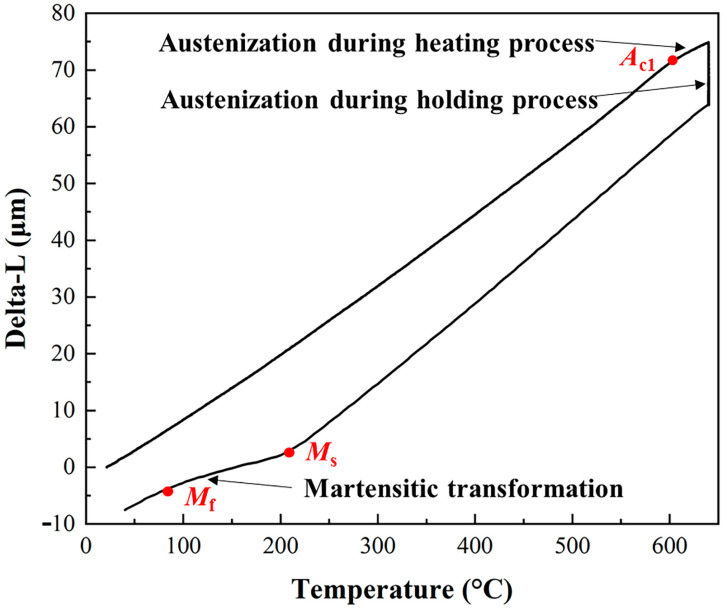
Dilatometric curve of 00Cr13Ni4Mo steel after tempering at 640 °C.

The volume fraction of reversed austenite depends on the austenite transformation in the tempering heating and holding stage and the martensitic transformation in the cooling stage. When austenite undergoes phase transition and transforms into martensite, one cell is transformed into two, and excess C is dissolved in the lattice, which enlarges the martensite lattice, and the volume of the material expands under the combined action of these two factors. In contrast, when martensite is converted to austenite, the volume is reduced. On the expansion curve, the volume expansion or contraction caused by martensitic transformation and austenite transformation are shown, i.e., the linear relationship between temperature and elongation will be broken by the volume change of the sample during the heat treatment. Therefore, the temperature of the phase transformation can be judged from the point of slope change on the thermal expansion result curve. The dilatometric curve of 00Cr13Ni4Mo steel after tempering at 640 °C is shown in Figure 3, showing the behavior of austenitization during the heating and holding process and martensitic transformation during the cooling process. The volume of the sample increases linearly with the temperature below the initial austenization temperature (*A*_s_). When the temperature exceeds *A*_s_, martensite begins to reverse into austenite, leading to a decrease in the volume expansion rate of the sample. Martensite continues to reverse into austenite during the holding process, resulting in a rapid decrease in the sample volume. In the cooling stage, the volume of the sample shows a linear decline with the temperature that is higher than the initial temperature for martensitic transformation (*M*_s_). When the temperature drops below *M*_s_, some reversed austenite grains formed during the heating and holding process gradually undergo martensitic transformation, resulting in a slower volume shrinkage rate of the sample. When the temperature drops to the final temperature for martensitic transformation (*M*_s_), the phase transition in the sample ends and the shrinkage rate of the sample volume recovers.

**Figure 4:**
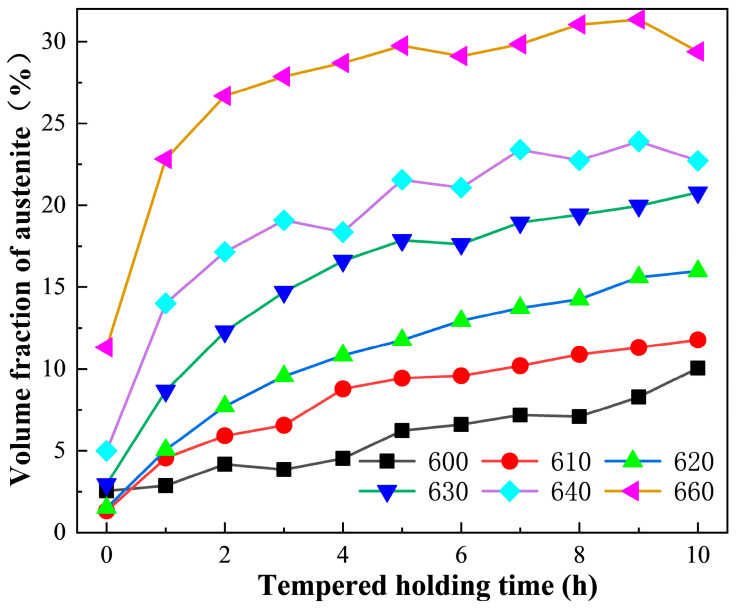
The variation curves of the reversed austenite volume fraction with holding time during the tempered holding stage.

The variation curves of the reversed austenite volume fraction with holding time during the tempered holding stage are shown in Figure 4. It can be seen that the volume fraction of reversed austenite first increases rapidly and then increases slowly with the tempering time; in particular, the reversed austenite tempering at 640 °C and 660 °C gradually increases to the saturation state with the excessive tempering time. This indicates that the growth rate of reversed austenite gradually slows down, or even stops with thermodynamic equilibrium. Furthermore, the higher the tempering temperature, the shorter the time taken for austenite transformation to reach thermodynamic equilibrium. Under the same holding time, the volume fraction of the reversed austenite of 00Cr13Ni4Mo steel is positively correlated with the tempering temperature.

**Figure 5:**
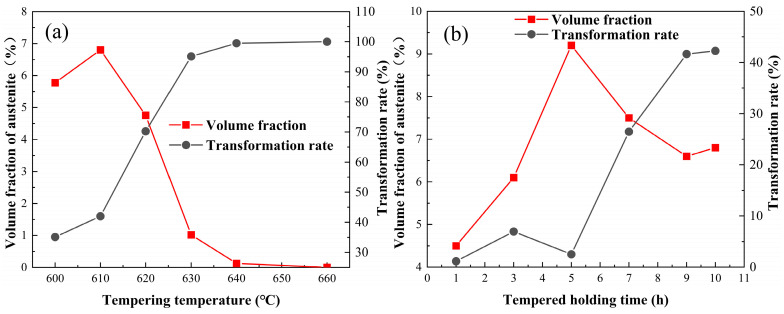
The volume fraction and the corresponding martensitic transformation rate of reversed austenite in 00Cr13Ni4Mo steel after tempering: (**a**) holding at different tempering temperatures for 10 h, and (**b**) tempering at 610 °C with different isothermal holding times.

Figure 5 shows the volume fraction and the corresponding martensitic transformation rate of reversed austenite in 00Cr13Ni4Mo steel after different tempering processes. The volume fraction of reversed austenite at room temperature continues to increase with the increase in the tempering temperature in the range of 580~610 °C, reaching a peak value of 6.8% at 610 °C, and then decreasing to 0% at 660 °C, as shown in Figure 5a. Furthermore, it can be seen that there is a huge difference between the volume fraction of reversed austenite in the tempering holding stage and that after cooling to room temperature. The volume fraction of reversed austenite is lowest in the holding stage of tempering at 610 °C and becomes highest after cooling to room temperature. For the case of tempering at 660 °C, the volume fraction of reversed austenite decreases from 31% in the tempering holding stage to 0% at room temperature. The ratio of martensitic transformation in austenite at different tempering temperatures is counted, as shown in Figure 5a. With the increase in the tempering temperature, the transformation rate of reversed austenite in the cooling stage increases rapidly and reaches 100% at 660 °C. This indicates that the tempering temperature has a significant effect on the thermal stability of reversed austenite. When the tempering temperature is 600 °C, the thermal stability of the reversed austenite is higher, so the ratio of transformation into martensite is lower. Then, the thermal stability of reversed austenite gradually weakens as the tempering temperature increases, making it more prone to martensitic transformation during the cooling process. Therefore, the volume fraction of reversed austenite shows a parabola-like variation with the increase in the tempering temperature.

Figure 5b shows the volume fraction and the corresponding martensitic transformation rate of reversed austenite in 00Cr13Ni4Mo steel after tempering at 610 °C with different holding times. It can be seen that the volume fraction of the reversed austenite increases firstly with the extension of the holding time, reaching a maximum of about 9.2% at 5 h, followed by a decreasing trend with a value of about 6.8% at 10 h. Similarly, the thermal stability of the reversed austenite affected by the holding time is analyzed using the transformation rate, as shown in Figure 5b. The transformation rate of reversed austenite is very small when the tempering time is less than 5 h, and with the extension of the tempering time, the transformation rate increases sharply and reaches 42.3% at 10 h. This indicates that the reversed austenite has good thermal stability below 5 h, and most of the austenite can be retained at room temperature. However, as the tempering holding time continues to extend, the thermal stability of the reversed austenite gradually weakens, making it more prone to martensitic transformation during the cooling process. Therefore, the volume fraction of reversed austenite shows a parabola-like variation with the increase in the isothermal holding time.

**Figure 6:**
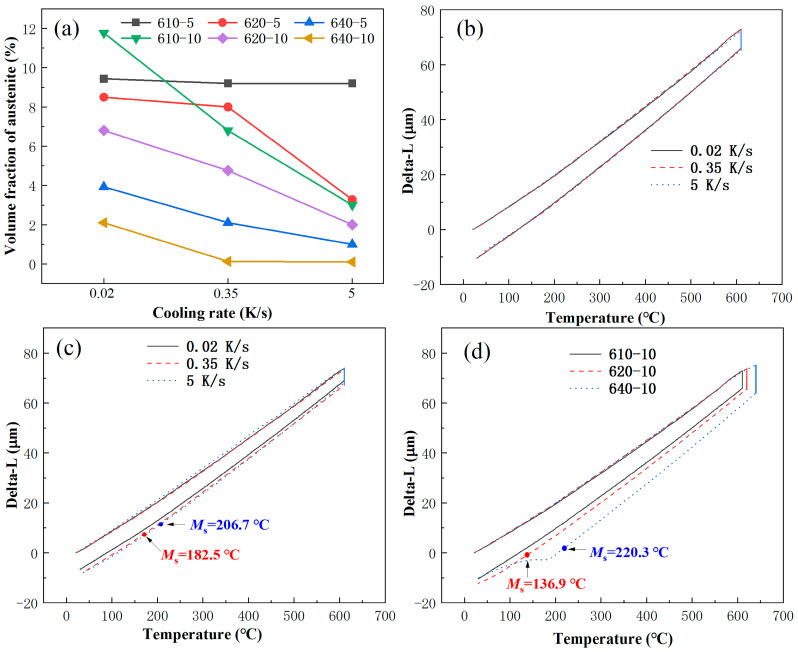
(**a**) Volume fraction of reversed austenite in 00Cr13Ni4Mo steel after tempering with different cooling rates. (**b**) Dilatometric curve of 610-5 with different cooling rates. (**c**) Dilatometric curve of 610-10 with different cooling rates. (**d**) Dilatometric curve of samples tempering at different temperatures with a holding time of 10 h.

The volume fraction of reversed austenite in 00Cr13Ni4Mo steel after tempering at 610, 620 and 640 °C with different cooling rates (0.02, 0.35 and 5 K/s) is shown in Figure 6a. It is found that the volume fraction of reversed austenite decreases with the increase in the cooling rate. This indicates that the cooling rate has a significant impact on the thermal stability of reversed austenite. In particular, the volume fraction of the reversed austenite in samples after tempering at 610 °C with a holding time of 5 h (610-5) changes little with the increase in the cooling rate. The *M*_s_ point on the thermal expansion curves can also support this interesting phenomenon, as shown in Figure 6b. No martensitic transformation occurs in 610-5 tempering at different cooling rates, which means that almost 100% of the reversed austenite formed at a high temperature remains at room temperature. Reversed austenite in 610-5 has the best thermal stability. Then, with the extension of the tempering time to 10 h, the thermal stability of the reversed austenite decreases gradually, and it is also affected by the cooling rate, which decreases rapidly with the increase in the cooling rate. As shown in Figure 6c, the *Ms* point of samples tempering at 610 °C for 10 h (610-10) increases with the increase in the cooling rate. Like 610-5, almost 100% of the reversed austenite formed in 610-10 remains at room temperature, with no obvious martensitic transformation at a cooling rate of 0.02 K/s. Furthermore, the *Ms* point at the same cooling rate increases with the tempering temperature, as shown in Figure 6d. The combined effect of the tempering temperature and holding time on thermal stability is much greater than that of the cooling rate. This also provides a new idea for retaining 100% of the austenite formed at a high temperature to room temperature.

### 3.2. Evolution of Reversed Austenite and the Effect of Carbide Precipitation during the Intercritical Tempering

**Figure 7:**
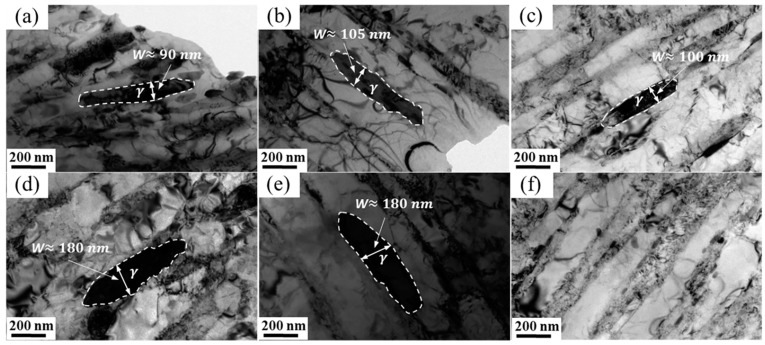
TEM morphologies of reversed austenite in 00Cr13Ni4Mo steel after tempering at different temperatures with holding times of 10 h: (**a**) 600 °C, (**b**) 610 °C, (**c**) 620 °C, (**d**) 630 °C, (**e**) 640 °C, and (**f**) 660 °C.

Figure 7 shows the TEM morphologies of reversed austenite in 00Cr13Ni4Mo steel after tempering at different temperatures with a holding time of 10 h. The morphology of the reversed austenite is flaky, mainly distributed at the boundary of the martensite lath, and its size is significantly affected by the tempering temperature. The reversed austenite in samples tempered at 600 °C, 610 °C, and 620 °C appears as long flakes with a size of about 100 nm in width, as shown in Figure 7a, Figure 7b and Figure 7c, respectively. The reversed austenite in samples tempered at 630 °C and 640 °C also presents as a long flake with a large size of about 180 nm in width, as shown in Figure 7d and Figure 7e, respectively. Although large-sized austenite is detected in Figure 7e, there is also a small amount of small-sized austenite decomposed from large-sized austenite in a sample tempered at 640 °C. Furthermore, only lath martensite can be detected in the sample tempered at 660 °C (Figure 7f), and all the reversed austenite transforms into the martensite during the cooling process (Figure 7a). Therefore, it can be considered that with the increase in the tempering temperature, the reversed austenite gradually coarsens, but its thermal stability also gradually decreases, resulting in a complete transformation into martensite after tempering at a higher temperature, which is consistent with Figure 4 and Figure 5.

**Figure 8:**
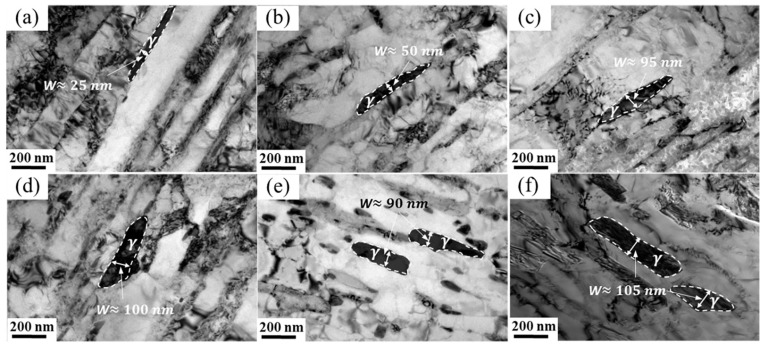
TEM morphologies of reversed austenite in 00Cr13Ni4Mo steel after tempering at 610 °C with different holding times: (**a**) 1 h, (**b**) 3 h, (**c**) 5 h, (**d**) 7 h, (**e**) 9 h, and (**f**) 10 h.

**Figure 9:**
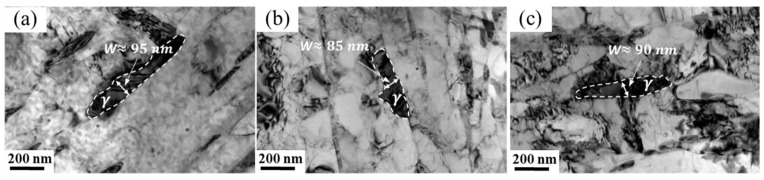
TEM morphologies of reversed austenite in 00Cr13Ni4Mo steel after tempering at 620 °C for 5 h with different cooling rates: (**a**) 0.02 K/s, (**b**) 0.35 K/s, and (**c**) 5 K/s.

Figure 8 shows the TEM morphologies of reversed austenite in 00Cr13Ni4Mo steel after tempering at 610 °C with different holding times. The morphology of the reversed austenite after tempering with a short holding time is a slender flake, as shown in Figure 8a and Figure 9b. It is known in Figure 5b that the volume fraction of the reversed austenite is the highest when the tempered holding time is 5 h, which means that the thermal stability of the austenite is the best at this time, and the width of the austenite flake further increases to 95 nm, as shown in Figure 8c. Then, as the tempering time continues to extend, the width of the austenite does not change significantly. It should be noted that the morphology of some austenite after tempering for 9 h and 10 h is blocky, as shown in Figure 8e,f. The thermal stability of some austenite decreases under a prolonged holding time and gradually spheroidizes into blocks. The periodization of the austenite flake decreases the interfacial energy barrier and forces the reversed austenite into martensitic transformation, resulting in a decrease in the volume fraction of the reversed austenite, as shown in Figure 5b.

As shown in Figure 6a, the volume fraction of reversed austenite is affected by the cooling rate. The morphologies of the reversed austenite in 00Cr13Ni4Mo steel after tempering at 620 °C for 5 h with different cooling rates are comparatively analyzed in Figure 9. However, there is not much difference in the morphology and size of the reversed austenite with different cooling rates. This means that the morphology of the reversed austenite is not affected by the cooling rate, and may be mainly attributed to the effect of the tempering temperature and holding time. 

**Figure 10:**
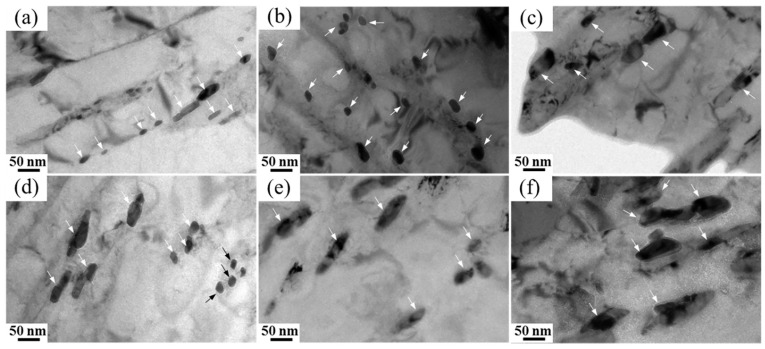
TEM morphologies of M_23_C_6_ carbides formed in 00Cr13Ni4Mo steel after tempering at different temperatures: (**a**) 600 °C, (**b**) 610 °C, (**c**) 620 °C, (**d**) 630 °C, (**e**) 640 °C, and (**f**) 660 °C.

The thermal stability of reversed austenite is related to the quantity and morphology of precipitated carbide. Figure 10 shows the TEM morphologies of the precipitated carbide formed in 00Cr13Ni4Mo steel at different tempering temperatures. The carbides dispersed in the martensite matrix are produced in the intercritical tempering process of the steel, which is M_23_C_6_ in type [9,16,24]. As shown in Figure 10a,b, the M_23_C_6_ carbides produced by tempering at 600 °C and 610 °C are fine in size and dispersed on the martensite matrix. With the increase in the tempering temperature, the quantity and size of M_23_C_6_ carbides gradually increase, and the maximum is at the tempering temperature of 660 °C, as shown in Figure 10f. It can be inferred that the presence of M_23_C_6_ carbides weakens the thermal stability of reversed austenite.

In thermodynamics, the martensitic phase transformation can only be produced when the chemical free energy $\Delta G_{ch}$ is negative [25]. However, there are non-chemical free energies $\Delta G_{nch}$, including interfacial and strain energies. Therefore, the thermodynamic criterion for the martensitic transformation is as follows [26]:

(1) $$\Delta G_{ch}+\Delta G_{nch}\leq 0$$

$\Delta G_{crit}$ is the critical chemical driving force for martensitic transformation and $\left|\Delta G_{crit}\right|-\left|\Delta G_{ch}\right|$ is a measure of austenite thermal stability. The concentration of austenitic solute elements is an important factor in determining the chemical driving force for the martensitic transformation [27]. For the Fe-Ni-C system, it can be expressed by the following equation [28]:

(2) $$\left|\Delta G_{crit}\right|-\left|\Delta G_{ch}\right|=|2x_{Ni}\left(1-x_{Ni}\right)-7.32x_{C}|(T-M_{s})$$

Here, $x_{Ni}$ and $x_{C}$ are the molar fractions of Ni and C in the alloy. From Equation (2), it can be seen that the thermal stability of austenite in the Fe-Ni-C system is influenced by both Ni and C. In low-carbon martensitic stainless steels, the content of Ni is generally much higher than that of C. Therefore, the value of $\left|2x_{Ni}\left(1-x_{Ni}\right)-7.32x_{C}\right|$ increases with the enrichment of Ni, and the austenite thermal stability increases. Also, $\left(T-M_{s}\right)$ is a factor that affects the thermal stability, C, Ni and Mn usually lead to a decrease in $M_{s}$ [29,30], and according to Equation (2), these elements increase the austenite thermal stability. Element C in the martensite matrix diffuses into the reversed austenite and aggregates to form M_23_C_6_ carbides during the intercritical tempering process. Furthermore, with the increase in the tempering temperature and the extension of the holding time, the quantity and size of carbides gradually increase. In this process, the precipitation and growth of M_23_C_6_ in reversed austenite dilute the carbon concentration in reversed austenite. The decrease in carbon concentration leads to a gradual decrease in the thermal stability of reversed austenite. In addition, the shear transformation of reversed austenite to martensite needs to overcome a certain interfacial energy resistance. When the specific surface area of reversed austenite is large, the greater the interfacial energy resistance to be overcome, which means that the difficulty of martensitic transformation is greater. In this case, the thermal stability of reversed austenite is better. At the initial stage of tempering, the morphology of the reversed austenite is mainly slender flake with a large specific surface area, which then becomes blocky with the increase in the tempering temperature and holding time. The blocky austenite has a lower specific surface area than the flaky austenite. Therefore, the blocky reversed austenite suffers less resistance induced by interfacial energy when it transforms back to martensite. Consequently, the blocky austenite can more easily transform to martensite during the cooling process, resulting in a decrease in the volume fraction of the reversed austenite after the intercritical tempering, as shown in Figure 5. These analyses indicate that in addition to the chemical composition of the reversed austenite, other factors influence the thermal stability of the reversed austenite and the final amount of reversed austenite obtained after the intercritical tempering. 

The above discussion sheds some light on the parabola-like variation in the volume fraction of reversed austenite with the tempering temperature and isothermal holding time. However, the experimental results of the cooling rate in this paper show unique phenomena. Generally, a faster cooling rate induces a larger degree of supercooling, enhancing the driving force for the martensitic transformation. Therefore, more reversed austenite retransforms into martensite during the cooling process at a higher cooling rate. Hence, the amount of reversed austenite decreases with an increase in the cooling rate. However, the effect of a slow cooling rate on the thermal stability of reversed austenite is often neglected, so it is not clear how to retain 100% of the reversed austenite formed at a high temperature at room temperature. Figure 7 shows that a reasonable tempering temperature and holding time can achieve this goal under a slow cooling rate. Although the martensitic transformation during cooling is a shear-type phase transition, carbon, as an interstitial atom, may diffuse and redistribute during the transformation. In particular, when the Ms point is high, the diffusion coefficient of the C atom is large, and the effect of its redistribution on the phase transition cannot be ignored. When the cooling rate is slow, the martensitic transformation may be accompanied by the redistribution of carbon atoms from the supersaturated martensitic matrix to the reversed austenite, improving the thermal stability of the reversed austenite and thereby reducing the *M*_s_ point, as shown in Figure 6c. In addition, the carbon diffusion from the decomposition of M_23_C_6_ carbides during the cooling process is also affected by the cooling rate. In the cooling process, C atoms decomposed from M_23_C_6_ carbides are polarized at defects such as dislocation to form a “C atom gas mass”. The size of the C atom gas mass is related to temperature and gradually increases with the decrease in temperature. At high temperatures, the atoms have a strong diffusion ability and mainly exist in the form of carbides, so the size of the “C atomic gas mass” is also relatively small. Then, the atom diffusion ability weakens with the decrease in temperature, and the tendency of the C atom to segregate increases gradually. During slow cooling, the larger “C-atom air mass” plays a stabilizing role in the reversed austenite. During rapid cooling, M_23_C_6_ carbides are not decomposed, and the formation of “C atom gas mass” is inhibited, which reduces the shear resistance of martensitic transformation, so the thermal stability of reversed austenite is reduced. These conclusions are consistent with the results shown in Figure 6.

### 3.3. Modeling the Kinetics of Reversed Austenite in the Tempered Holding Stage

The kinetics of austenite formation during the tempering holding phase were simulated using the JMAK model. The model treats the nucleation rate and the radial growth rate as constant, and the phase fraction (*X*) as a function of time (*t*) for the phase in which nucleation and growth occur simultaneously is given by the following equation [31]:

(3) $$X=1-\exp\left(-kt^{n}\right)$$

where *k* is the temperature-dependent dynamic constant and *n* is the Avrami index. Since a portion of austenite is already present in the organization at the beginning of the tempering holding stage, and since holding austenite in the two-phase zone does not completely transform it, the kinetic equation for the isothermal transformation of reversed austenite can be expressed as follows:

(4) $$X_{r}=\left(X_{eq}-X_{r}^{0}\right)\left[1-\exp\left(-kt^{n}\right)\right]+X_{r}^{0}$$

where $X_{r}$ is the volume fraction of austenite at any point in the tempering holding stage, $X_{eq}$ is the volume fraction of austenite at the tempering temperature when thermodynamic equilibrium is reached, and $X_{r}^{0}$ is the volume fraction of austenite already present in the organization at the beginning of the holding stage. The results of the experiments for each process can be brought to fit to obtain the values of *n* and *k*, as shown in Table 2.

**Figure 11:**
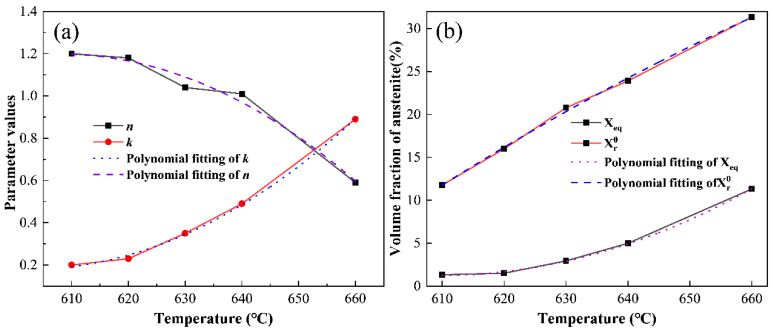
Polynomial fitting curves of *n*, *k*, $X_{eq}$ and $X_{\gamma }^{0}$. (**a**) *n* and *k*; (**b**) $X_{eq}$ and $X_{\gamma }^{0}$.

To correlate each parameter with the process, the *n* value, *k* value, $X_{eq}$, $X_{r}^{0}$ and tempering temperature for the scatter plot are shown in Figure 11; The values of each parameter are quadratically related to the tempering temperature, so a polynomial was fitted using a y = A_1_ + A_2_ × T + A_3_ × T^2^ type equation. The results of the fitted parameters are shown in Table 3. The values are brought into Equation (4) to obtain a mathematical model of the kinetics of austenite formation in the tempering holding stage, which can predict the reversed austenite content at any moment of the holding stage.

### 3.4. Modeling the Kinetics of Reversed Austenite in the Cooling Stage

During cooling, the martensitic phase transformation does not occur when the material is cooled to a temperature where the free energies of the two phases are equal, and the phase transformation can only occur when the material is subcooled to a temperature below the M_S_ point, which therefore represents the temperature at which the minimum driving force is required for the transformation from austenite to martensite. The martensitic phase transformation becomes a non-diffusive phase transformation, and the non-diffusive phase transformation is temperature-controlled, corresponding to a temperature-dependent phase fraction. In this paper, the empirical equations for the martensitic phase transformation established by Buchmayr et al. are used, and these are given in Equation (5) [32]:

(5) $$F_{M}=F_{a}(1-exp(-\alpha (M_{S}-T_{q})))$$

where $F_{M}$ is the amount of martensitic transformation, $F_{a}$ is the remaining austenite when the martensitic transformation starts, $M_{S}$ is the temperature at which the martensitic transformation starts, $T_{q}$ is the instantaneous temperature during the cooling process, and α is the martensitic transformation constant. The modification of the KM formula can be obtained by combining the experimental results of this material:

(6) $$X_{\gamma }^{T}=X_{\gamma }-(X_{\gamma }-X_{\gamma }^{25})[1-exp(-\alpha (M_{s}-T))]$$

where $X_{\gamma }^{T}$ is the austenite volume fraction at any time during the cooling process, $X_{\gamma }$ is the austenite volume fraction at the beginning of cooling, and $X_{\gamma }^{25}$ is the austenite volume fraction when cooling to room temperature. Above the M_S_ point, the austenite to martensite transition does not take place, so $X_{\gamma }$ is the austenite fraction at high temperatures, calculated from Equation (4), and brought to the corresponding holding time.

The values of parameters α and 1 calculated from the experimental data were correlated with the holding time and tempering temperature using the following equations:

(7) $$y=B_{1}+B_{2}t+B_{3}t^{2}$$

(8) $$B=C_{1}+C_{2}T+C_{3}T^{2}$$

where *t* is the holding time and *T* is the tempering temperature. *B* and *C* are the fitted parameters, with their specific values shown in Table 4, Table 5, Table 6 and Table 7.

M_S_ points are correlated with the holding time and tempering temperature using the following formula:

(9) $$y=B_{1}+B_{2}t$$

(10) $$B=C_{1}+C_{2}T+C_{3}T^{2}$$

where *t* is the holding time and *T* is the tempering temperature. *B* and *C* are the fitted parameters, with their specific values shown in Table 8 and Table 9.

The values of each parameter are brought into Equation (6) to obtain the variation curve of the austenite content with cooling temperature during the cooling process, and the austenite content can be predicted at any temperature during the cooling process. When the temperature is taken to room temperature, the room temperature austenite content can be calculated, so based on the cooling model in Equation (6), the correlation equations of each parameter with the holding time, tempering temperature and the holding model in Equation (4) can be brought in to obtain the model for the final prediction of the room-temperature austenite content.

### 3.5. Verification of Model

To ensure the rationality of the model, the in situ XRD experiment at 635 °C for 10 h was compared with the calculated results to verify whether the change in reversed austenite in the calculated tempering and holding stage was consistent with the experiment. By bringing 635 °C into Equation (4), the theoretical relationship between the volume fraction of reversed austenite and the tempering holding time at the tempering temperature of 635 °C can be obtained. Equation (11):

(11) $$Y=18.37\times \left(1-\exp\left(-0.42\times x^{1.04}\right)\right)+3.93$$

where *Y* is the volume fraction of contravariant austenite and *x* is the holding time.

**Figure 12:**
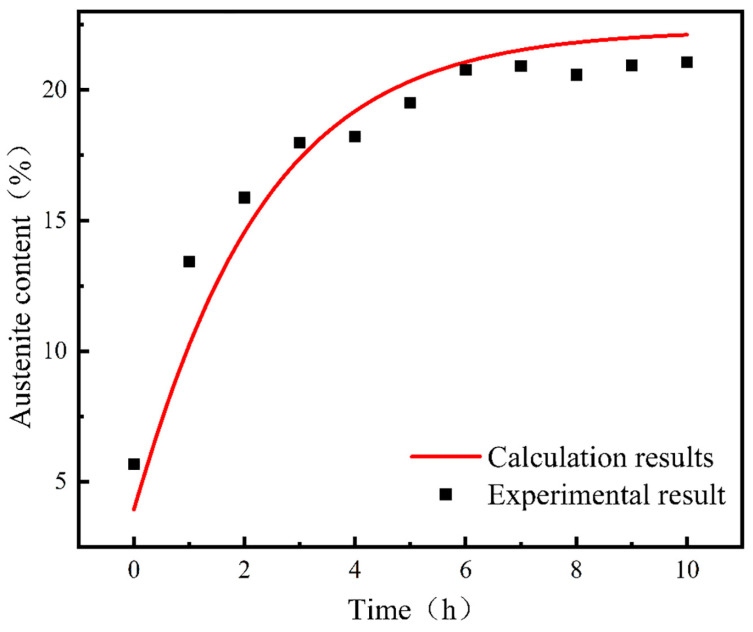
Comparison between calculation curve and experimental results of the austenite content in 00Cr13Ni4Mo steel after tempering at 635 °C.

The calculation results and experimental results are shown in Figure 12. It can be seen that the gap between the theoretical calculation results and experimental results is no more than 5%, which indicates that the theoretical model established is reasonable.

For the mathematical model of the process and the reversed austenite content, the experimental results of different holding times at 610 °C were selected for comparison and verification. Because the cooling method is air cooling, the cooling rate is 0.35 K/s, and 610 °C is substituted into Formula (4) to obtain high-temperature data; then, a tempering temperature of 610 °C and cooling rate of 0.35 K/s are put into Formula (6) to calculate the reversed austenite volume fraction formula for different holding times at 610 °C, as shown in Equation (12):

(12) $$\begin{array}{cc} Y=\left(11.96-1.37\right) & \times \left(1-\exp\left(-0.19\times x^{1.17}\right)\right)+1.37\\ & -(\left(11.96-1.37\right)\times \left(1-\exp\left(-0.19\times x^{1.17}\right)\right)+1.37\\ & -\left(0.3+3x-0.26x^{2}\right))\times (1\\ & -\exp(-\left(\left(-2.15E^{-18}\right)+0.0015x+\left(8E^{-6}\right)x^{2}\right)\\ & \times \left(\left(92.98+5.82x\right)-10\right))) \end{array}$$

where *Y* is the volume fraction of contravariant austenite and *x* is the holding time.

**Figure 13:**
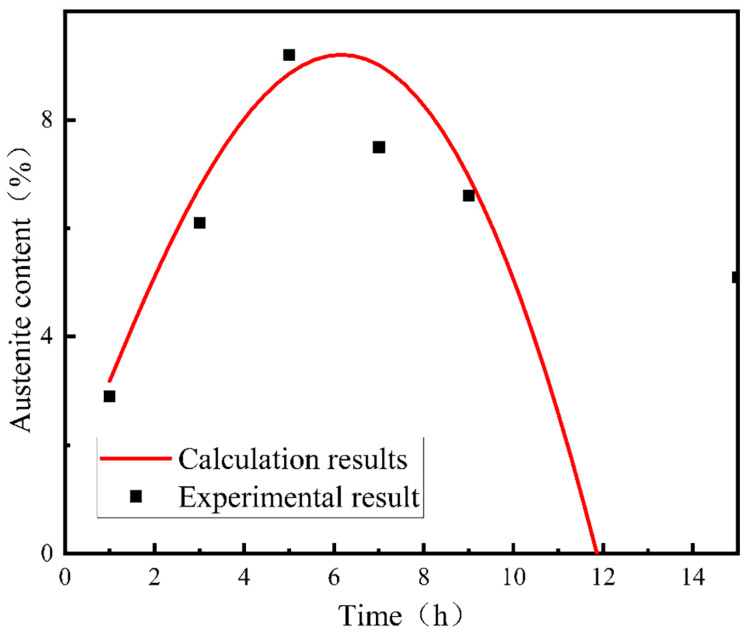
Comparison of calculation curves and experimental results of the austenite content in 00Cr13Ni4Mo steel after tempering at 610 °C for different holding times.

The calculated theoretical value of the reversed austenite volume fraction at different holding times at 610 °C is compared with the experimental results at different holding times at 610 °C, as shown in Figure 13. It can be seen that when the tempering holding time is less than 10 h, the difference between the theoretical calculation results and the experimental results is not more than 5%. When the tempering holding time exceeds 10 h, these parameters are not within the effective range of the equation, and the results are far from the experimental results. So, in the effective range, the theoretical model established is reasonable.

## 4. Conclusions

1. The in situ X-ray diffraction experiments showed that the volume fraction of reversed austenite at high temperatures increases with the tempering temperature. The reversed austenite content at room-temperature increases with the tempering temperature first to 6.8% at 610 °C and then decreases to 0% at 660 °C, and increases with the holding time first to 9.2% at 5 h and then decreases to 6.8% at 10 h. The reversed austenite content at room temperature continues to decrease as the cooling rate increases. The results show that the reversed austenite content in martensitic stainless steel can be further controlled by controlling the tempering process parameters, and the specific reversed austenite content of martensitic stainless steel can be obtained.
2. Through TEM observation, the results show that with the increase in the tempering temperature or increase in the holding time, M_23_C_6_ will further form and grow, and a certain amount of carbon elements in the reversed austenite will be consumed by the formation and growth of M_23_C_6_, so that the carbon concentration in the reversed austenite will continue to decline, resulting in the continuous deterioration of thermal stability. Furthermore, the reversed austenite size increases from 100 nm to 180 nm with an increasing tempering temperature, from 25 nm to 95 nm with an increasing holding time, and does not change significantly with the cooling rate. Meanwhile, the morphology of austenite changes from fine lath to block with the extension of the tempering time.
3. Based on the traditional JMAK model, a modified model of the high-temperature evolution of reversed austenite in 0013Cr4NiMo steel was established through in situ X-ray diffraction experiments. The volume fraction change equation of reversed austenite at a high temperature was obtained, and the calculated values were in good agreement with the experimental values. Based on the traditional KM model, the contravariant austenite structure evolution model of 0013Cr4NiMo steel was modified and established through a thermal expansion experiment. The contravariant austenite volume fraction change equation was obtained, and the calculated values were in good agreement with the experimental values.

## Figures and Tables

**Table 1:** Chemical composition of 00Cr13Ni4Mo steel (wt.%).

Element	C	Si	Mn	Cr	Ni	Mo	Fe
Mass fraction (%)	0.026	0.39	0.65	12.44	4.13	0.50	81.86

**Table 2:** Parameter values of different tempered temperatures.

	610 °C	620 °C	630 °C	640 °C	660 °C
*n*	1.20	1.18	1.04	1.01	0.59
*k*	0.20	0.23	0.35	0.62	0.89

**Table 3:** The parameters for the fitting curves.

	A_1_	A_2_	A_3_
*n*	−81.30	0.27	−2.21 × 10^−4^
*k*	77.09	−0.256	2.13 × 10^−4^
$X_{eq}$	−724.62	1.97	−1.25 × 10^−3^
$X_{\gamma }^{0}$	1476.53	−4.48	3.97 × 10^−3^

**Table 4:** The fitting results of the relationship between α and the tempering holding time.

	B_1_	B_2_	B_3_
610 °C	−2.15 × 10^−18^	1.50 × 10^−3^	8 × 10^−6^
620 °C	−4.01 × 10^−18^	2.92 × 10^−3^	−1.04 × 10^−4^
640 °C	−1.20 × 10^−17^	6.32 × 10^−3^	−2.92 × 10^−4^

**Table 5:** The fitting results of the relationship between B_1_, B_2_, B_3_, and the tempering temperature (α).

	C_1_	C_2_	C_3_
B_1_	−2.41 × 10^−15^	8.02 × 10^−18^	−6.69 × 10^−21^
B_2_	2.15 × 10^−1^	−8.38 × 10^−4^	8 × 10^−7^
B_3_	2.95 × 10^−2^	−8.5 × 10^−5^	6 × 10^−8^

**Table 6:** The fitting results of the relationship between $X_{\gamma }^{25}$ and the tempering holding time.

	B_1_	B_2_	B_3_
610 °C	0.3	3	−0.26
620 °C	−0.12	2.72	−0.22
640 °C	−0.07	0.83	0.081

**Table 7:** The fitting results of the relationship between B_1_, B_2_, B_3_ and the tempering temperature ($X_{\gamma }^{25}$).

	C_1_	C_2_	C_3_
B_1_	617.8	1.97	1.57 × 10^−3^
B_2_	−828.8	2.73	−2.24 × 10^−3^
B_3_	80.78	−0.264	2.15 × 10^−4^

**Table 8:** The fitting results of the relationship between *M*_S_ and the tempering holding time.

	B_1_	B_2_
610 °C	18.97	18.58
620 °C	199.46	3.58
640 °C	216.06	5.91

**Table 9:** The fitting results of the relationship between B_1_, B_2_ and the tempering holding time (M_S_).

	C_1_	C_2_	C_3_
B_1_	−48,951.02	153.6	−0.12
B_2_	6520.62	−21.05	0.017

## Data Availability

All data included in this study are available upon request from the corresponding author.
